# Qualitative comparison of elemental concentration in soils and other geomaterials using FP-XRF

**DOI:** 10.1371/journal.pone.0268268

**Published:** 2022-05-20

**Authors:** Pranjal Singh, Manoj Datta, G. V. Ramana, Sanjay Kumar Gupta, Tabarak Malik

**Affiliations:** 1 Department of Civil Engineering, Indian Institute of Technology, New Delhi, Delhi, India; 2 Department of Biochemistry, College of Medicine & Health Science, School of Medicine, University of Gondar, Gondar, Ethiopia; Texas A&M University System, QATAR

## Abstract

Field portable X-ray fluorescence (FP-XRF) has tremendous potential in geoenvironmental engineering as a qualitative assessment tool. Identification of the elevated concentrations of the selected elements (Cr, Cu, Mn, Ni, Pb, and Zn) in various geomaterials like soil-like-material (SLM), incinerated bottom ash (IBA), construction and demolition waste (CDW), zinc tailings (ZT) and fly ash (FA) was performed by FP-XRF and compared with the local soil–Delhi silt. Comparably higher concentrations (mg/kg) of Cr (401.0), Cu (499.0), Pb (532.0), Zn (608.0) in SLM, Cr (195.0), Cu (419.0), Ni (93.0), Pb (931.0), Zn (771.0) in IBA and Cr (195.0), Cu (4000.0), Pb (671.0), Zn (7122.0) in ZT were observed. CDW and FA showed similar concentrations range as in local soils. FP-XRF was also used in-situ on local soil at 11 sites to examine its ability to identify the elements with significant variations in concentrations. The results showed high variability in Cl and S concentration values across the 11 sites attributed to the changing moisture content and dissolved salts. The concentration range for the remaining elements were similar at all sites. The verification of the detected elements through visual inspection of the spectrum was also carried out.

## Introduction

FP-XRF is a widely used as a screening tool for the detection of elements in the contaminated soils. It is a rapid, mobile, easy-to-use, non-destructive, and cost-effective alternative to the conventional laboratory methods like AAS, ICP-AES, and ICP-MS for qualitative analysis [1]. It can measure total elemental concentration, independent of chemical form, of elements ranging from Al (atomic number = 13) to U (atomic number = 92) [2]. FP-XRF was initially developed for commercial use in testing lead-based paints, precious metals, airborne particulates, glass composition, archaeology, and mining [3]. Its application expanded to environmental soil testing at the start of the 20^th^ century. Currently, US EPA [4], USDA [5], and ISO [6] have their guidance documents for the use and application of FP-XRF in soils.

The fundamental theory of X-ray fluorescence is well documented in books, research articles, and manuals [7–9]. Nevertheless, a summary of the working principles of FP-XRF is presented herewith. When an incident X-ray photon strikes an atom, an electron may get ejected out if the energy of the X-ray is greater than the binding energy of that electron. Subsequently, another electron from a higher energy shell moves to the vacant shell of the ejected electron. A fluorescent X-ray photon is released with energy equal to the difference between the two shells. This fluorescent photon strikes the FP-XRF detector, which registers the count and energy of such photons. The latter is unique to an element and is used to identify the elements present in the sample matrix, whereas the former represents the concentration. Finally, an X-ray spectrum is generated with characteristic peaks, which can be used for qualitative and quantitative estimation. Additionally, filters are used between the source and the sample to increase the sensitivity towards a specific range of elements. The filter suppresses the background X-rays generated from the source to enable a better signal-noise ratio [10]. FP-XRF can be used in-situ or after applying sample preparation methods. In situ analysis requires only rough manual homogenization and flattening of the exposed surface and can only be used for qualitative estimation [11]. On the contrary, quantitative analysis can be carried out post the sample preparation process, which includes oven-drying or air drying, homogenization through rifling or quartering, pulverization through ball milling and finally sieving.

Various researchers have made significant efforts to validate the elements detected by FP-XRF and their corresponding concentrations based on the correlation with laboratory results [12–14]. But instrument-based validation/calibration is time-consuming and is only required for definite data generation [15]. However, the most practical use of FP-XRF is as a qualitative screening tool, particularly to identify the elements of concern or the hotspots of contamination. For the identification of elements of concern, a background material must be used for reference. But it may not be necessary that the matrix of material in question is the same as the background material, and since the output of the FP-XRF is dependent on the type of matrix, the same calibration should not be used for both materials. The default factory setting of the instrument has separate calibration for matrices with trace concentration such as soil, called Compton Normalisation calibration, and high concentrations such as metal ores, called Fundamental Parameters calibration [3]. Most case studies previously used FP-XRF on only one type of material using a particular type of calibration [16–18]. Its performance on different matrices has not been discussed. Therefore, it is relevant to study its response to materials with vastly different matrices such as zinc tailings, soil-like material, etc., to ascertain if it qualitatively identifies the elements with elevated concentrations. In this study, FP-XRF is used with hybrid calibration on five geomaterials and compared with local soil. A hybrid method automatically determines the type of calibration based on the concentration of the elements if the matrix type is unknown [8].

Elements reported by FP-XRF are based on the definition of the limit of detection (LOD), i.e., three times the standard deviation, representing the deviation of photon counts per second from the mean counts per second detected over the entire analysis time [19]. Since it is a statistical estimation, it may give false positives, especially if the concentration is near the LOD [20]. In these cases, it becomes necessary to verify the elements reported by the instrument by visual inspection of the spectrum [21]. Most of the case studies on FP-XRF rely solely on the instrument’s values, and this aspect of visual inspection is not discussed [22, 23]. Hence, a discussion regarding a verification by visual inspection of the spectrum in a blank silica sample is made, which is missing in the available previous literature.

There is a significant difference in the variability of FP-XRF results in a controlled environment undergoing extensive sample preparation versus in the field with no sample preparation [11]. This is due to the natural heterogeneity of soil in terms of density, size of particles, water content, organic content, etc. [9]. Very few studies have evaluated the performance of FP-XRF when used directly at the site [24, 25]. Moreover, its ability to report the variations in concentration in the field arising due to reasons apart from natural heterogeneity is also not studied. In this study, FP-XRF is used in-situ at 11 sampling points having local soil to study this aspect.

The objectives of this study are as follows: (1) verifying the authenticity of reported elements in a blank sample using visual inspection of the spectrum (2) identifying the elements with significantly elevated concentrations in five commonly encountered geomaterials using FP-XRF (3) highlighting the variation in elements arising apart from natural heterogeneity in the soil using in-situ use of FP-XRF. These three mentioned aspects are critical for the establishment of FP-XRF as a qualitative tool for contamination studies.

## Materials

### Blank sample, local soils, and geomaterials

A blank sample of SiO_2_ was analyzed with a certified concentration of 99.999% to check the reliability of the elements detected by the FP-XRF internal software. For the comparative study between the geomaterials and local soil, a total of eight samples were analyzed (three local soils and five geomaterials). The local soils were Badarpur sand (BS), Delhi silt (DS), and Yamuna sand (YS). The geomaterials were soil-like material (SLM), incinerated bottom ash (IBA), construction and demolition waste (CDW), zinc tailings (ZT), and fly ash (FA). The samples were prepared by oven drying (at 105° C, 24 h), pulverization using ceramic mortar and pestle, crushing in a steel ball mill (for 4 h), sieving through a 75-micron sieve, and then analyzed in XRF cups containing 10–15 g sample. Five samples for each material were tested, and average concentrations were reported.

#### Badarpur sand

BS is the locally quarried sand classified as SP as per the Unified Soil Classification System (USCS). The coefficient of uniformity (C_u_) and curvature (C_c_) are 1.93 and 0.97, respectively. The average grain size (D_50_) is 0.246 mm, and the effective grain size (D_10_) is 0.45 mm. The unit weight ranges from 13.97 kN/m^3^ to 16.28 kN/m^3^. The SEM analysis reveals the angular nature of the particles with sharp edges. The angle of shearing resistance (Φ) is 42.5° based on direct shear tests [26].

#### Delhi silt

DS is an alluvial soil lying in the Indo-Gangetic plains around Delhi, India. The significant component is silt (35%-80%) followed by sand (10%-43%) with minimal clay content (3%-7%). The sand particles are subangular to subrounded shaped, and silt particles are similar to ground quartz. The plasticity index varies from 4.5%-8.0%. The effective angle of shearing resistance (Φ’) ranges from 31–34° calculated using a conventional triaxial test [27].

#### Yamuna sand

YS is collected from the banks of the river Yamuna in Delhi, India. It is classified as SP with little gravel and fine content with C_u_ = 2.60 and C_c_ = 0.72. The average grain size (D_50_) = 0.48 mm. The angular shaped soil majorly contains quartz (40%) and feldspar (40%) with carbonates (18%) and mica (1%-2%) as minor constituents. The angle of shearing resistance is 39.5° [28].

#### Soil-like material

SLM is less than 4.75 mm fraction of the mined waste from the three dumpsites in Delhi: Okhla, Ghazipur, and Bhalswa. It is roughly 55%-65% of the total municipal solid waste segregated by trommels at the dumpsite itself. Total dissolved salts, sulfates, chlorides, and bicarbonates are higher in SLM than in local soil. Also, heavy metals like As, Cr, Cu, Ni, Pb, and Zn are 3–15 times higher. Organic content is ranging between 4.8%-24.5% [29].

#### Incinerated bottom ash

IBA is the residue of the refused derived fuel (RDF) burned at 850–1000°C and is collected from waste-to-energy plants located in Okhla and Narela, Delhi. Silt and clay, sand, and gravel-sized particles range between 10%-13%, 53%-63%, and 23%-40%, respectively. The chemical composition includes SiO_2_ (55%), CaO (15%), Al_2_O_3_ (11%), Fe_2_O_3_ (5%), K_2_O (2%), MgO (3%) P_2_O_5_ (1.6%) and Na_2_O (1%) with sulfates and chlorides as 1% and 0.6%-0.9%, respectively. Organic matter is 3%-5.7% [30].

#### Construction and demolition waste

This material is obtained from CDW processing plants in Burari, Shastri park, and Noida located in Delhi NCR. The output of these plants consists of recycled concrete aggregate (RCA) (10%), recycled aggregate (RA) (55%), and a fraction below 0.075 mm (35%). RA is a mixture of concrete, brickbats, and other materials. The sand-sized fraction contributes around 90% of the material, with the remaining being less than 0.075 mm. C_u_ and C_c_ range from 3.81–5.80 and 1.08–1.13, respectively. MDD achieved through the standard proctor test was 16.9–17.2 kN/m^3^. The peak angle of shearing resistance ranges from 34.8° to 48.7° in dense and loose conditions [31].

#### Zinc tailings

ZT are the residues from the metal extraction process from ores which are finally deposited in a tailings dam. It consists of fractions that are unfeasible to extract from the ores. Due to the presence of metals, the specific gravity is higher than soils, equal to 2.91. The bulk density is 1.49 kN/m^3^. D_50_ and D_10_ are equal to 29.92 μm and 4.75 μm. ZT has mostly irregular to angular-shaped particles with sharp edges. The mineralogical composition of ZTs show high presence of SiO_2_ (33%), CaO (17%), Fe_3_O_3_ (16%), Al_2_O_3_ (12%), SO_3_ (10%) and MgO (6.43%), followed by minor minerals such as K_2_O (1.73%), P_2_O_5_ (1.08%), Na_2_O (0.49%) and TiO_2_ (0.37%) [32].

#### Fly ash

Fly ash is obtained from coal ash ponds of the thermal power plant situated in Delhi. Coal ash consists of the finer fly ash collected in the electrostatic precipitators and coarser bottom ash deposited at the bottom of the furnace. Fly ash contains 20% sand-sized and 80% silt-sized particles and is classified as SM as per USCS. It consists of predominantly smooth hollow spherical shaped particles and consequently has a lower specific gravity of 2.08. The maximum dry unit weight is 13.9 kN/m^3^ with a minimum void ratio of 0.49. The average grain size (D_50_) = 32 mm. The angle of shearing resistance is 31° calculated using direct shear tests [33].

### Instrument used

A Niton XL3t GOLDD+ FP-XRF by Thermo Scientific is used in this study. It contains a 50 kV and 200 μA excitation source with Ag anode. The analysis was carried out in three modes: ‘Main’, ‘Low’, and ‘High’ for 60 seconds each. The ‘Main’ mode is not optimized for any specific range of elements, but reasonable sensitivity is achieved for all elements. ‘Low’ mode uses a copper filter optimized for low range elements (with fluorescent X-ray energy ranging between 0–5 keV), and ‘High mode’ uses a molybdenum filter for high range elements (5–28 keV) [8]. A qualitative assessment of the spectrum was carried out to identify all the characteristic and artifact peaks. The characteristic peaks were identified using Kα and Kβ with the intensity of Kα/Kβ roughly equal to 5–7 [4]. For heavier elements, only Lα and Lβ peaks were identified with a ratio of 1:1 [21]. A hybrid model of the calibration was used for quantification in which Compton calibration was used for trace elements, and Fundamental Parameters calibration was used for high concentration elements.

## Methodology

### Blank sample

**Fig 1:**
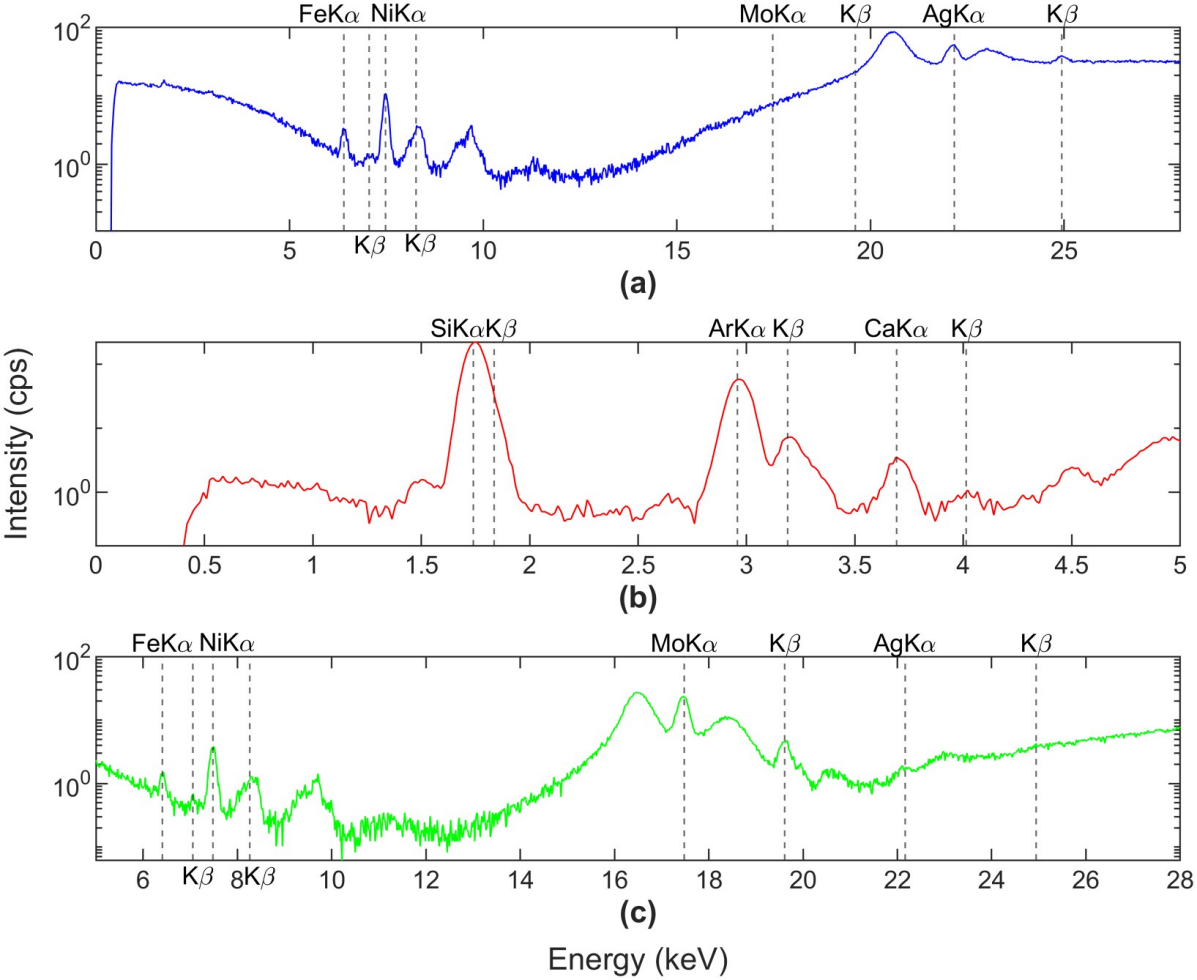
Fluorescent x-ray spectrum of a blank SiO_2_ sample. (a) spectrum in ‘Main’ mode without any filters. (b) and (c) are spectra in ‘Low’ mode and ‘High’ mode using Cu and Mo filters, respectively.

Fig 1 shows the X-ray spectrum of the blank SiO_2_ sample, (a) representing the spectrum in ‘Main’ mode with no filters. As a result, the unfiltered Rayleigh and Compton’s peaks arising from the Ag anode in the source are visible. The Rayleigh peaks are generated due to elastic scattering of incident X-rays, whereas the Compton peaks result from inelastic scattering [21]. There is no loss in energy in elastic scattering; hence the peaks occur at the incident X-ray energy. Inelastic scattering, however, results in loss of energy due to which peaks are at slightly lesser energy than incident X-rays. Additionally, the characteristic peaks of Si, Ca, Fe and Ni are visible. The presence of Ca, Fe, and Ni can be due to contamination of the laboratory apparatus or the sample itself. But the intensity is below ten counts per second (cps), which is too low to affect the qualitative analysis significantly. Fig 1(b) and 1(c) correspond to ‘Low’ mode and ‘High’ mode optimized for low range (0–5 keV) and high range (5–28 keV). Si and Ca peaks are visible in the low range, along with Ar Kα and Kβ peaks. Ar characteristic peak is due to its presence in the air between the source and sample [34]. For the high range, Rayleigh and Compton peaks arising from the molybdenum filter are visible along with characteristic peaks of Fe and Ni. Based on the results from blank samples, elements were categorized into two groups: visually identified elements on the spectrum, elements reported by FP- XRF above the limit of detection (LOD). This comparison was used to validate the elements reported by FP-XRF.

### Local soils and geomaterials

A comparative evaluation of the composition of the local soils was carried to observe the natural variation of heavy metals. Subsequently, the concentration of elements of other geomaterials was compared with DS, and the outliers were identified. A subjective criterion was used to identify outliers as no single rule could be applied across all concentration range. The elements of concern for the analysis were Cr, Cu, Mn, Ni, Pb, and Zn. Particularly for fly ash, two analyses were carried out: one with water content = 15% and the other oven-dried (at 105°C) to analyze the effect of moisture content on the XRF readings.

The samples of Delhi silt and geomaterials were also analyzed for the same heavy metals using AAS. Duplicate samples were prepared using microwave-assisted aqua regia (HCl + HNO_3_) digestion. 50 ml diluted samples were prepared using 3 ml HCl and 9 ml of HNO_3_ on 0.5 g of sample. Some samples were further diluted for elements having concentrations above the calibration range of AAS.

### In-situ sampling

**Fig 2:**
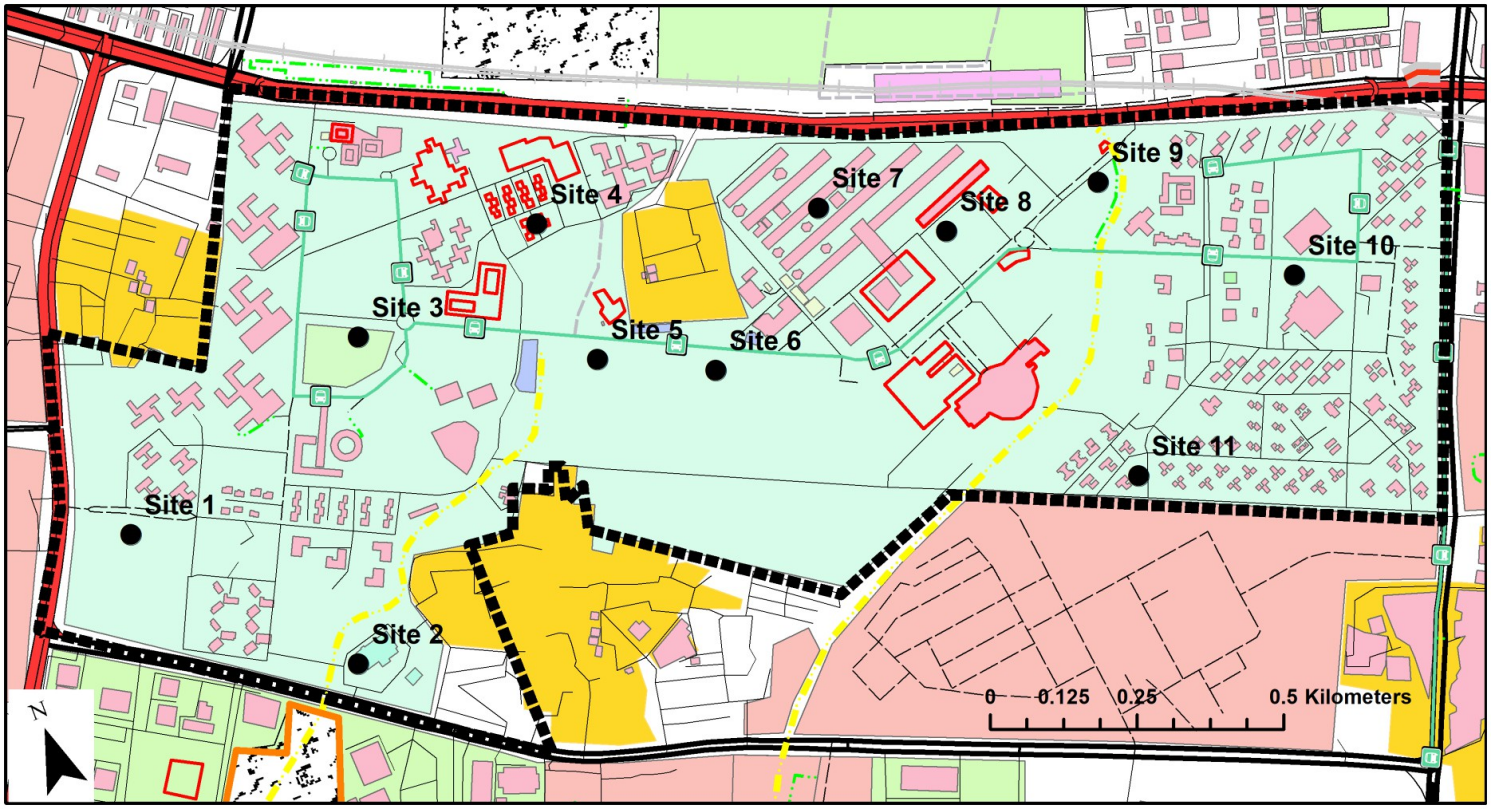
Sampling locations in IIT Delhi campus (India) for in-situ analysis using FP-XRF. Source: OpenStreetMap^®^.

The variation of in-situ results of FP-XRF was evaluated by analyzing samples taken from eleven locations on the campus of IIT Delhi (India). The location of sampling points is shown in Fig 2. The campus of IIT Delhi, located in the national capital of New Delhi, is spread across 325 acres. At each sampling point, a total of five readings with an analysis time of 60 seconds per filter, spaced half a meter apart, were taken to get an average reading. The moisture content varied across all sampling sites from dry to damp conditions. A high moisture content decreases the intensity of characteristic X-ray peaks, decreasing the concentration values. At even 20% water content, the decrease will be approximately 20% which is satisfactory for qualitative assessment [35].

## Results and discussion

### Blank sample

The elements visually identified using the spectrum were Si, Ca, Fe and Ni. However, the instrument reported 18 elements above LOD: Al (311 mg/kg), Si (410000 mg/kg), Fe (83 mg/kg), Ni (32 mg/kg), Zn (9 mg/kg), Cd (15 mg/kg), K (136 mg/kg), V (17 mg/kg), Mn (111 mg/kg), Co (32 mg/kg), Ag (5 mg/kg), Sn (25 mg /kg), Sb (52 mg/kg), Te (105 mg/kg), Cs (39 mg/kg), Ba (288 mg/kg), Ce (32 mg/kg), and Pr (41 mg/kg). Ca peaks were visible in the spectrum but were undetected by FP-XRF. It may be due to shorter analysis time as the standard deviation increases as the time is reduced. Hence, it is evident that FP-XRF cannot be solely relied upon for detection but should be supplemented by visual identification of the spectrum [21].

### Comparison of local soils

**Fig 3:**
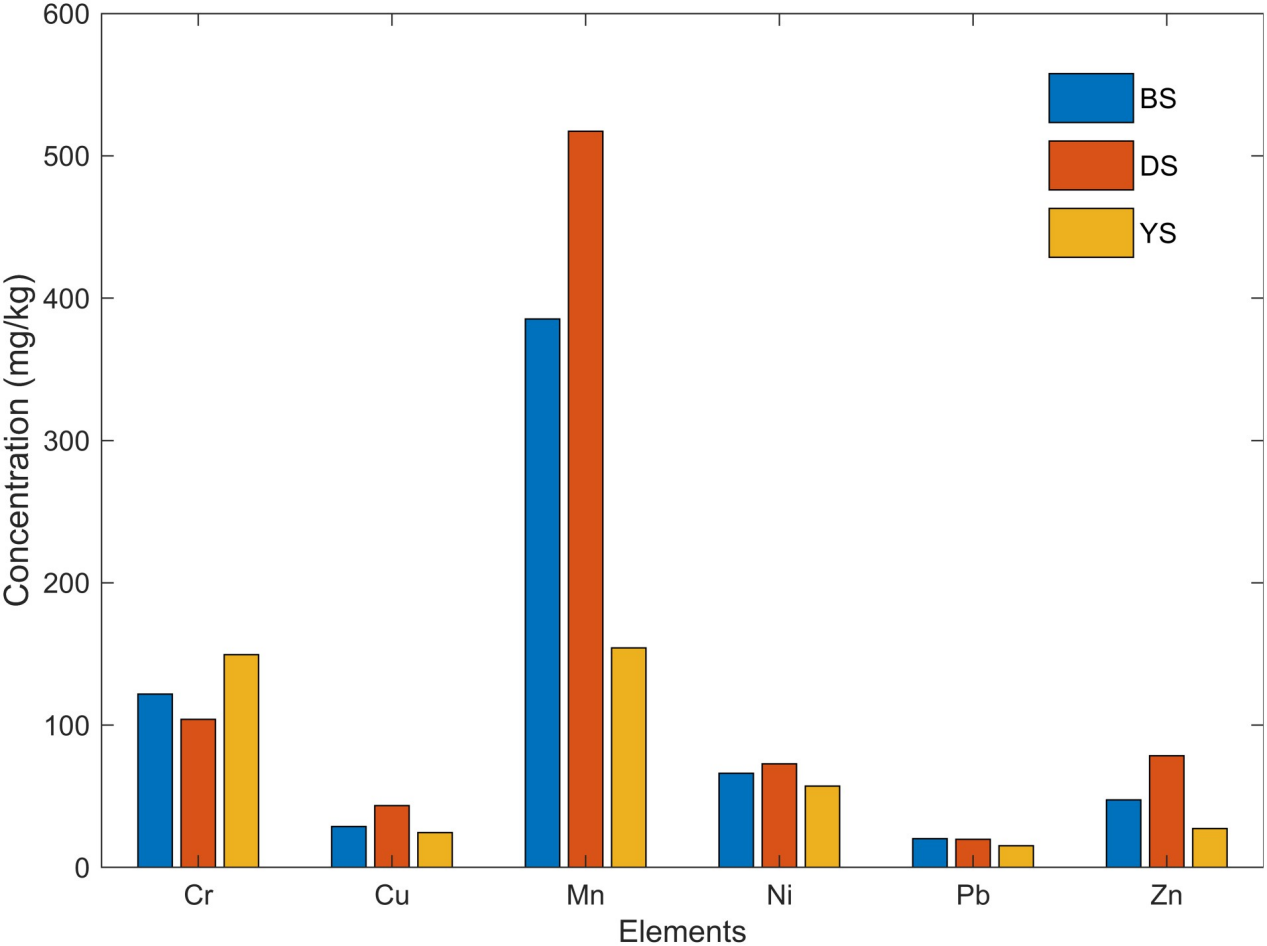
Comparison of elemental concentration in local soils: Badarpur sand, Delhi silt, and Yamuna sand.

Fig 3 shows the relative concentration of heavy metals in local soils: Badarpur sand (BS), Delhi silt (DS), and Yamuna sand (YS). All elements except Mn show minimal variability in the three soils. Mn concentration is lowest in YS, followed by BS, and highest in DS. YS appears to be the ‘cleanest’ in terms of heavy metal concentration due to its sedimentary nature. However, since the difference between concentrations is of the order of 10 mg/kg, this inference must be verified by laboratory analysis. The instrument also detected As, but it was not reported since no characteristic peak was visible in the spectrum. All the six heavy metals were also confirmed by Chen et al. [36], who conducted an elemental analysis of background soil in Beijing, China using laboratory methods. The reported mean concentrations were Cr (61 mg/kg), Cu (23 mg/kg), Mn (582 mg/kg), Ni (27 mg/kg), Pb (27 mg/kg) and Zn (74 mg/kg). These concentrations support the results of FP-XRF and show that the natural presence of heavy metals in local soil is similar across the globe. Somani et al. [29] calculated the aqua regia digested heavy metals in DS using ICP-MS, which were lower than the reported values in this study. The reported concentrations were: Cr (36–43 mg/kg), Cu (14–15 mg/kg), Mn (108–112 mg/kg), Ni (20–21 mg/kg), Pb (6–7 mg/kg) and Zn (44–67 mg/kg). These lower values are expected as aqua regia may not completely digest the heavy metals, whereas FP-XRF provides total elemental concentration [37]. In this study also, AAS values were lesser than FP-XRF for most of the elements. However, for some elements like Pb and Zn, AAS showed higher values due to the analytical error of FP-XRF or the sampling variability. Singh [38] conducted a heavy metal analysis on YS with the AAS method. The reported concentrations were Cr (394 mg/kg), Cu (275 mg/kg), Mn (695 mg/kg), Ni (159 mg/kg), Pb (76 mg/kg) and Zn (561 mg/kg). The concentrations are above their natural presence as all elements except Mn are categorized as moderately or highly polluted based on the geoaccumulation index.

### Comparison with Soil-Like Material (SLM)

**Fig 4:**
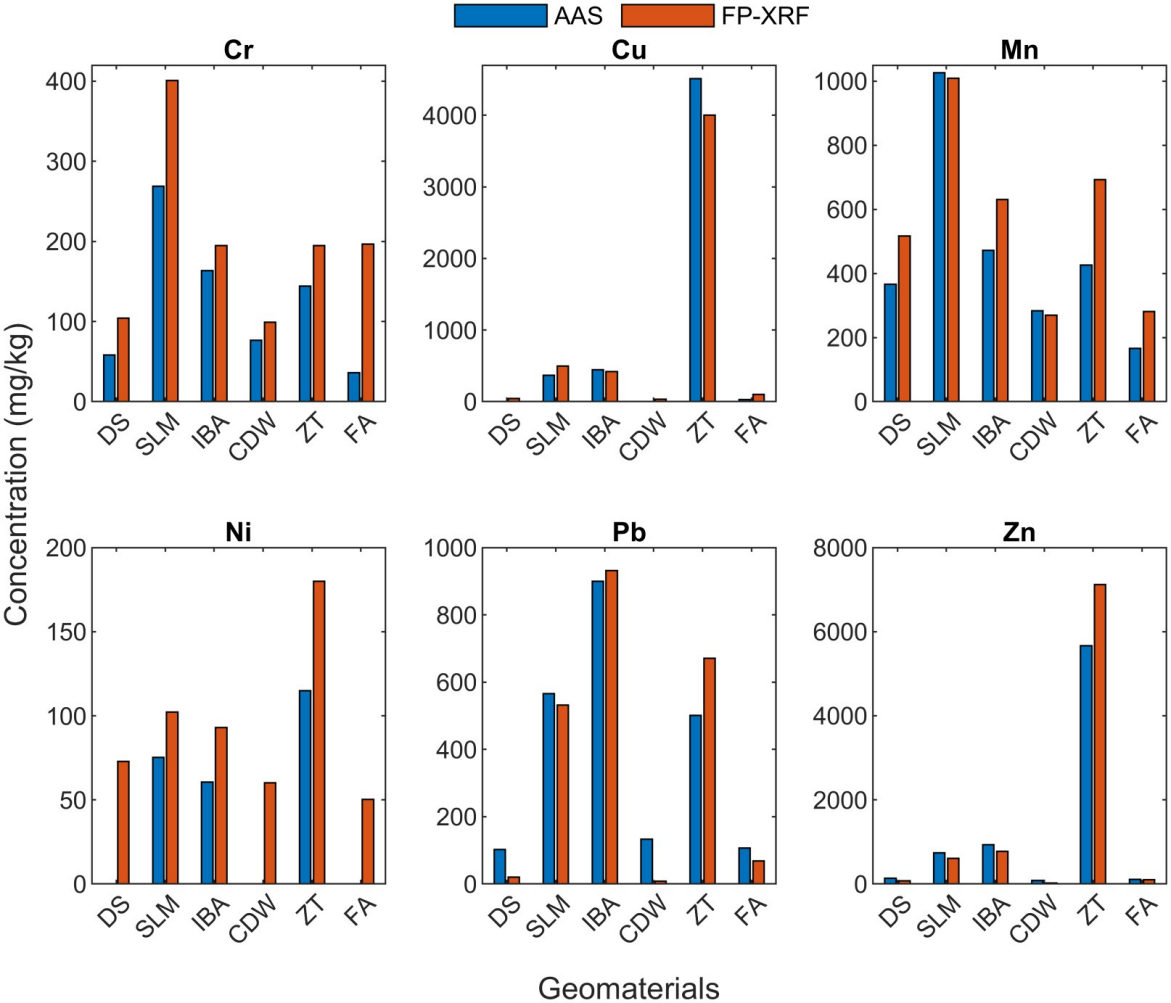
Comparison of elemental concentration of Delhi silt with geomaterials using FP-XRF and AAS.

Fig 4 shows the comparison of all the geomaterials with DS. For SLM, all heavy metals except Ni are higher than the local soil. There is a good agreement with AAS results except for Ni, which was not detected by AAS. Somani et al. [29] also reported a significantly higher concentration of Cr (89–339 mg/kg), Cu (140–574 mg/kg), Mn (198–408 mg/kg), Ni (26–97 mg/kg), Pb (27–333 mg/kg) and Zn (153–696 mg/kg) than DS.

### Comparison with Incinerated Bottom Ash (IBA)

Heavy metals in IBA except Pb are relatively lower than SLM as they may volatilize on conversion to their oxide forms. Still, the concentration of Cr, Cu, Pb, and Zn is higher than DS. Song et al. [39] reported Cr (80 mg/kg), Cu (1196 mg/kg), Mn (875 mg/kg), Pb (325 mg/kg) and Zn (1935 mg/kg) in IBA. These results do not agree with FP-XRF results as the elemental concentration in IBA is dependent on the composition of MSW. Since this study is conducted in South Korea, the nature of MSW is expected to be vastly different than in India.

### Comparison with Construction and Demolition Waste (CDW)

For CDW, the results are very similar to DS as the source of most of the construction materials are siliceous or argillaceous derived from natural soils and rocks. Hence, it has the potential to be reused as a geotechnical material. Ni was not detected by AAS similar to DS. Also, like DS, the concentration of Pb by AAS was higher than XRF. For the fraction of CDW less than 0.075 mm, Bianchini et al. [40] reported Cr (133 mg/kg), Ni (98 mg/kg), Pb (57 mg/kg) and Zn (144 mg/kg). Except for Ni, all values are close to what is reported by FP-XRF.

### Comparison with Zinc Tailings (ZT)

ZT are the leftovers after the refinement process of Zn ores to extract the metal [32]. Impurities in the form of other metals can be present in the ore. ZT is the most contaminated material in this study, with all heavy metals at significantly higher concentrations than DS, specifically Cu and Zn. Arsenic was also detected by FP-XRF but was not reported as its characteristic peaks are of similar energy as Pb and are not distinguishable on the spectrum. Usually, as a rule of thumb, if Pb concentration is ten times higher than the As concentration, then As concentration can be reliable, which is not valid in this case [20]. Elmayel et al. [32] reported Mn (450 mg/kg), Pb (3460 mg/kg) and Zn (10,217 mg/kg) in Pb-Zn tailings. Pb is expectedly higher as the ores were also used to extract Pb.

### Comparison with Fly Ash (FA)

**Fig 5:**
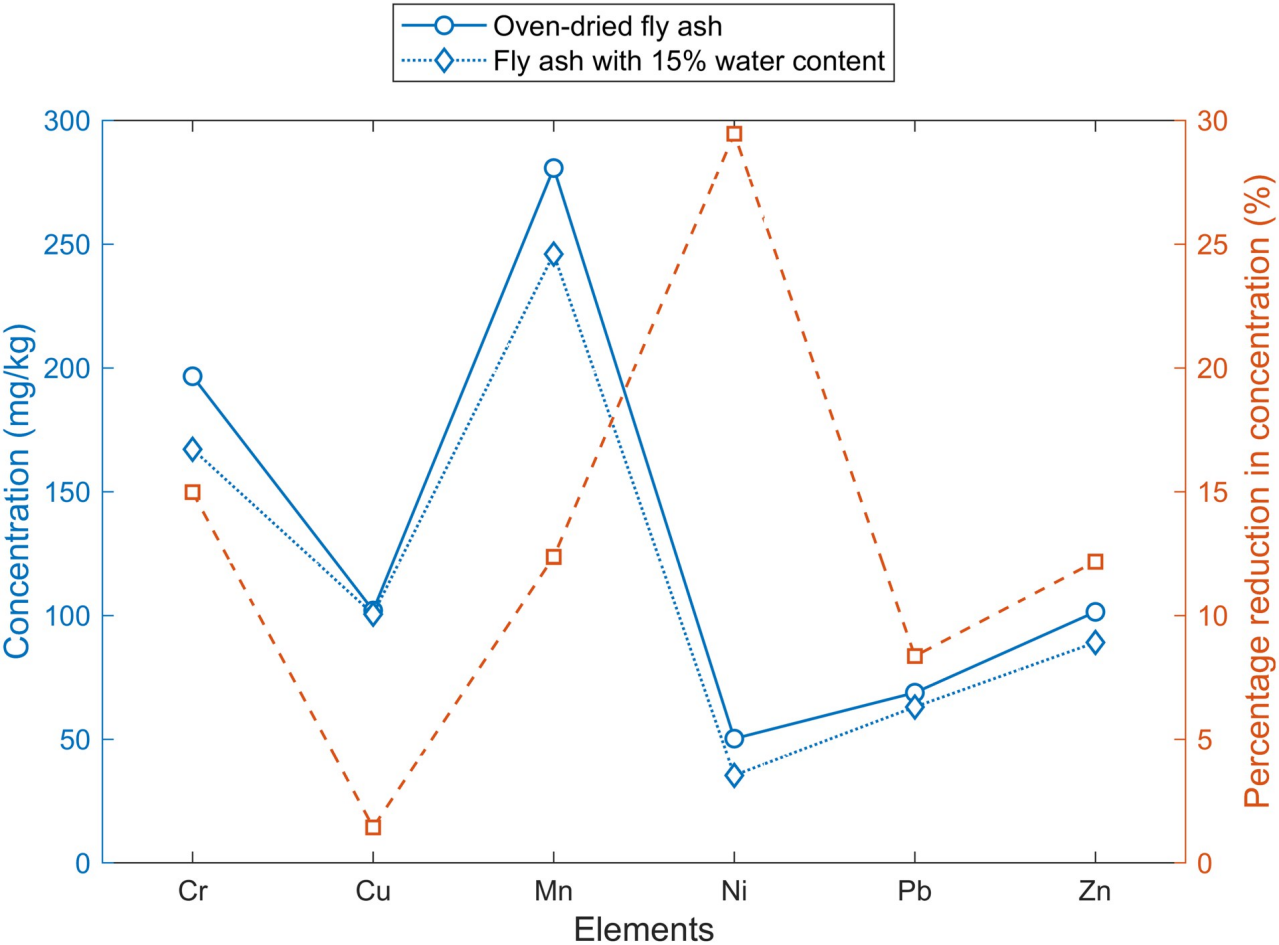
Comparison of elemental concentration in fly ash with water content = 15% and oven-dried fly ash.

FA has a similar elemental composition to DS with no metal at significantly elevated concentrations. Ni was not detected by AAS, and FP-XRF reported higher Cr than AAS. Tiwari et al. [41] reported Cr (51 mg/kg), Mn (308 mg/kg), Ni (52 mg/kg), Pb (23 mg/kg) and Zn (74 mg/kg). Another sample of fly ash with water content = 15% was examined with FP-XRF. Fig 5 shows that the moist sample showed decreased concentrations ranging from 2% to 30%. A similar decrease has been shown in the literature [35, 42].

### In-situ analysis

**Fig 6:**
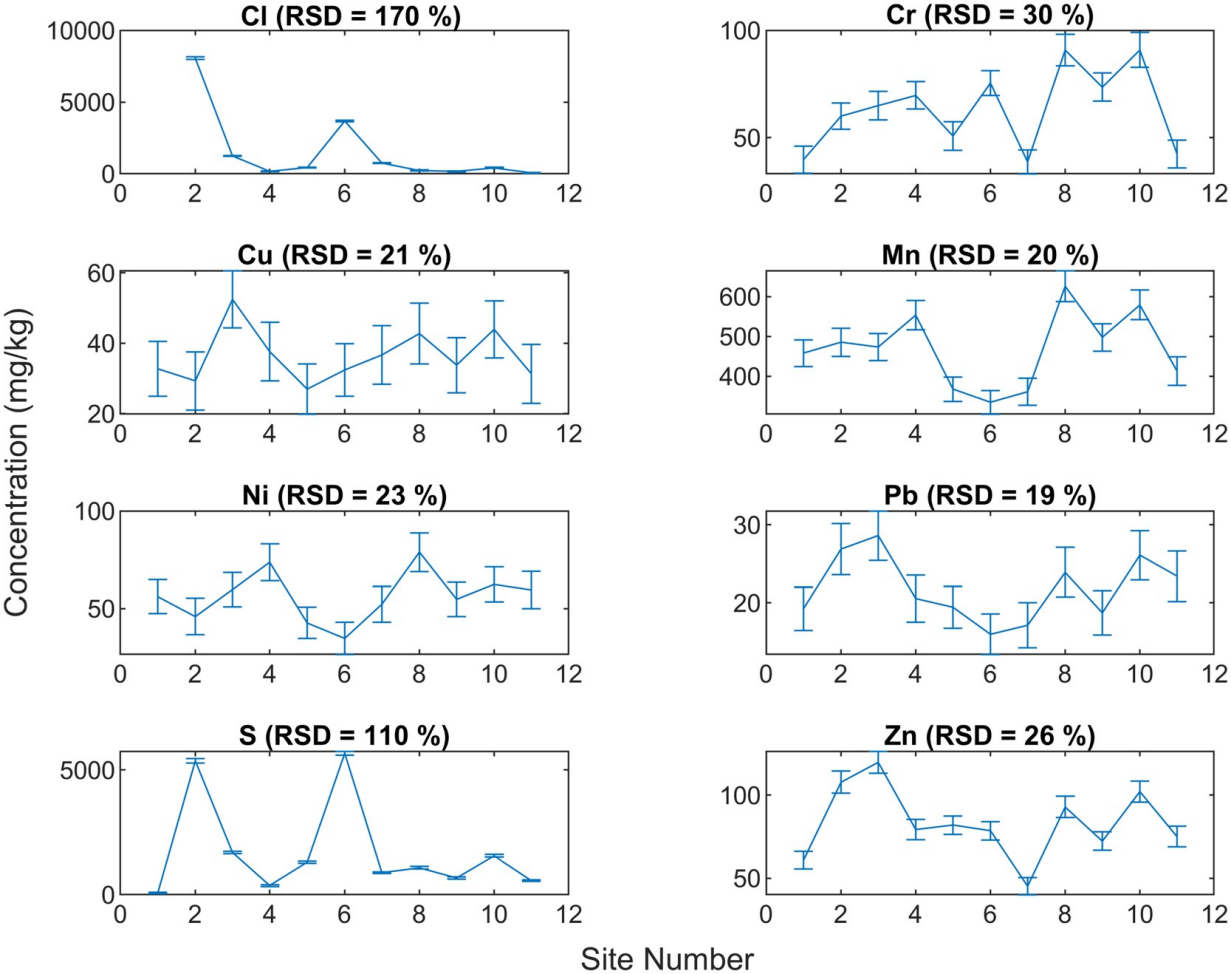
Elemental concentration in soils across eleven sites in IIT Delhi campus.

The results of in-situ tests carried out using FP-XRF at the eleven sites having DS are shown in Fig 6. The heavy metals detected were Cr, Cu, Mn, Ni, Pb and Zn ranging from 39–91 mg/kg, 29–52 mg/kg, 335–626 mg/kg, 35–74 mg/kg, 16–29 mg/kg, 45–120 mg/kg, respectively. The variability in heavy metal concentrations in local soils is less, and the relative standard deviation variation varies from 19% to 30%. This variability is due to natural, instrument, and sampling variation. However, certain elements such as Cl and S showed RSD of 177% and 110%. The sites containing higher S also reported higher Cl, showing their simultaneous presence. S and Cl may be present as dissolved salts in the soil moisture as the sites showing elevated values were in damp condition.

This study included the use of FP-XRF in the laboratory and the field. Some key points are highlighted regarding the operation of this equipment. The silicon detector in the instrument must be maintained at -25° C ± 5° C (maintained by liquid nitrogen) for the equipment to be operational. However, in many instances during the field study, FP-XRF stopped operating after a single analysis of 120s, especially when the outside temperature was greater than 35° C. Therefore, it is advised that FP-XRF only be used when the outside temperature is relatively low. Moreover, during the analysis in the lab, it was observed that the noise in the spectrum significantly reduced when the sample was densified by tapping 4–5 times before the analysis. Also, sometimes the FP-XRF is directly used on samples that are stored in HDPE bags. The polyethylene may attenuate the X-rays leading to more noise and decreased concentration values. The samples should first be analyzed with and without the HDPE bags to analyze its effect.

## Conclusions

The spectrum of blank silica sample showed peaks corresponding to Si, Ca, Fe and Ni, but the instrument reported the false presence of Al, Zn, Cd, K, V, Mn, Co, Ag, Sn, Sb, Te, Cs, Ba, Ce, Pr. This highlights that all the elements detected by FP-XRF, especially those near the LOD, must be verified by manual inspection of the X-ray spectrum. The check of Kα/Kβ or Lα/Lβ ratio should be used as confirmation of the detected elements. The FP-XRF was able to identify the contaminants with significantly elevated concentrations in SLM, IBA, and ZT. For SLM, Cr, Cu, Mn, Pb, and Zn were higher than the local soil–DS. For IBA, Cr, Cu, Pb, and Zn were higher. All heavy metals, specifically Cu and Zn, were extremely high in ZT. CDW and FA reported similar concentrations as in DS. Therefore, FP-XRF can qualitatively identify elevated elements in various other matrices, apart from its application in contaminated soil. A rapid screening by FP-XRF and further comparison with a background material followed by a targeted laboratory analysis can provide faster and economical results. The site data generated by FP-XRF was also able to depict the variation of Cl and S, which was maybe due to the variation in water content of the soil. Other elements showed similar results at all the sites as the natural heterogeneity of soils is not very significant. These results highlight that FP-XRF is a very effective tool for a quick estimation of hotpots at a contaminated site, where it can identify the variation in concentrations arriving due to reasons apart from the natural heterogeneity. This study shows the FP-XRF can be effectively used for qualitative assessments. However, several factors such as water content and organic matter introduce high variability in the results. Hence, the reliability of the qualitative evaluations must be researched further in these cases. Once these aspects are clearly understood, the application of FP-XRF can extend to other geomaterials such as municipal solid waste, where both water content and organic matter are relatively high. Also, several other techniques involving near-infrared (NIR) and mid-infrared (MIR) can be studied by combining with X-ray spectroscopy to achieve better detection and concentration values.

## Supporting information

S1 File (XLSX)
